# Is digital psychiatry really for all? A cross-sectional analysis from two randomized clinical trials

**DOI:** 10.47626/2237-6089-2024-0826

**Published:** 2025-11-05

**Authors:** Carolina Benedetto Gallois, Mairon Matheus Machado, Cauê dos Santos de Oliveira, Alice Castro Menezes Xavier, Carolina Blaya Dreher, Gisele Gus Manfro

**Affiliations:** 1 Universidade Federal do Rio Grande do Sul Hospital de Clínicas de Porto Alegre Faculdade de Medicina Porto Alegre RS Brazil Programa de Pós-Graduação em Psiquiatria e Ciências do Comportamento, Faculdade de Medicina, Hospital de Clínicas de Porto Alegre (HCPA), Universidade Federal do Rio Grande do Sul (UFRGS), Porto Alegre, RS, Brazil.; 2 Universidade Federal de Ciências da Saúde de Porto Alegre Faculdade de Medicina Porto Alegre RS Brazil Faculdade de Medicina, Universidade Federal de Ciências da Saúde de Porto Alegre (UFCSPA), Porto Alegre, RS, Brazil.

**Keywords:** Digital, psychiatry, vulnerable populations, internet access, technology, mental health

## Abstract

**Objective:**

Digital psychiatry holds promise for expanding accessibility to mental health treatment, but concerns exist regarding its inclusivity and the potential for exacerbation of digital exclusion among vulnerable populations. This study aims to evaluate the inclusivity of digital psychiatry research and interventions, and to explore their potential to worsen digital exclusion.

**Methods:**

We conducted a cross-sectional analysis of sociodemographic data from two clinical trials that utilize psychiatric online treatment modalities in Brazil. Participants were recruited nationwide through digital media platforms.

**Results:**

The sample comprised 224 individuals, predominantly female (95.1%) and Caucasian (71.87%) participants, with an average of 15.12 years of schooling. It was observed that White individuals were overrepresented compared to national averages (42.8%). Additionally, participants had a higher average number of years of schooling compared to the national average (10.1 years). Our analysis revealed a clear profile among psychiatric patients with access to and interest in digital interventions, predominantly younger, White, educated, and female individuals.

**Discussion:**

As digital therapeutic solutions advance, ensuring their inclusivity and accessibility for vulnerable individuals is crucial. Initiatives to promote digital inclusion and reassess participant recruitment strategies are needed to effectively address digital exclusion. By adopting multifaceted approaches, digital mental health care can be made more effective and accessible to all.

## Introduction

Access to mental health treatments has always been challenging, witnessing a demand that surpasses treatment capacity. In this context, digital psychiatry, defined as the integration of technologies within mental health care, emerges as an alternative to expanding treatment capacity and accessibility. Its aim is to promote well-being, aid in self-care, and facilitate early intervention or targeted treatment for specific mental illnesses.^1^ However, the actual extent of coverage of digital treatments needs to be evaluated. These technologies might further exacerbate the exclusion of vulnerable segments of the population from accessing mental health treatment, particularly for individuals with limited access to technology or difficulties in using it.

The term "digital exclusion" can be defined as an inability or unavailability of access to technological tools, such as computers, tablets, smartphones, and/or internet access.^2^ It mainly includes socially vulnerable individuals, such as the unemployed or low-income, illiterate or low-educated, the elderly, and residents of rural areas, especially from low and middle-income countries.^3^ The trend is for digital exclusion to grow with technological advancement, underscoring the need for a cautious perspective and preventive inclusion measures to prevent this gap from widening further.^4^

The aim of this study was to evaluate the capacity of digital psychiatry research in reaching and including vulnerable patient populations. By analyzing the sociodemographic characteristics of participants in two randomized clinical trials that used digital interventions as a treatment tool, we aimed to determine the extent to which digital interventions provide equitable access to individuals facing barriers to treatment access. Additionally, we sought to assess the level of inclusivity in studying these digitally vulnerable populations, indicating how thoroughly they are represented in digital psychiatry research. Our hypothesis is that the most vulnerable individuals, who already face significant challenges in accessing mental health treatments, may encounter even greater difficulties as psychiatry transitions to the digital environment. This potential disparity should be properly evaluated to implement effective inclusion and prevention measures.

## Methods

Our assessment is based on a cross-sectional analysis of the sociodemographic data collected from two clinical trials conducted by our research group in Brazil. These trials were entitled "Digital strategies for patients with chronic dermatoses with pruritus/dermatillomania" (Study 1) and "Digital interventions as an adjunct tool in the treatment of generalized anxiety disorder: a randomized clinical trial" (Study 2). They focused on utilizing online treatment modalities and employed a nationwide recruitment strategy through various digital media platforms, a methodology commonly adopted by conventional "non-digital" projects as well.

Both studies were developed between March 2021 and December 2023 and were properly registered in clinical trials (NCT04731389 and NCT05375851), approved by the ethics committee at the Hospital de Clínicas de Porto Alegre (CAAE: 37827820.6.0000.5327 and 54357621.7.0000.5327), and conducted in accordance with the principles outlined in the Declaration of Helsinki. All participants in the studies completed the consent form. The studies were conducted entirely online, including recruitment, interventions, and assessments and included participants from across the country. The assessment of the sociodemographic data was self-reported by the participants and conducted using the Brazil Economic Classification Criteria of 2015, facilitated through the REDCap platform.

The Study 1 included 163 patients with Skin Picking Disorder (SPD) who were randomly assigned to receive treatment with cognitive-behavioral therapy (CBT) delivered by a remote platform or to an active control group (psychoeducational videos on healthy living habits) for 4 weeks. Inclusion criteria were a diagnosis of SPD according to the DSM-5, being 18 years or older, proficiency in Brazilian Portuguese, and access to the internet. Exclusion criteria included acute mania, active psychotic episode, severe major depressive episode, and substance use disorder (except tobacco). The sample was recruited through media advertisements targeting profiles related to SPD on Brazilian Facebook^®^ and Instagram^®^. The sample size was calculated based on a previous study with 133 individuals that used remote CBT to treat SPD, aiming for a statistical power of 95%, an alpha error of 5%, and an estimated loss rate of 20%.

In Study 2, we selected participants with GAD diagnosis to examine the impact of adding psychoeducational videos and self-administered psychometric scales via mobile devices to the treatment with fluoxetine on clinical outcomes such as GAD-7 and HAM-A rating scales after 12 weeks. Inclusion criteria required participants to be 18 years or older, have GAD diagnosis according to DSM-5, and have access to the internet and a mobile phone or a tablet with internet connectivity. Exclusion criteria were individuals with psychiatric comorbidities more severe than GAD, those at risk of suicide, who were pregnant or breastfeeding, and those currently receiving or having received psychological or psychiatric treatment in the last three months. The sample was recruited through digital media advertisements by the Hospital de Clínicas de Porto Alegre (including their website and social media), as well as through social media accounts of team members. The sample size of 60 subjects was calculated with 80% power, a 5% significance level, and a standard deviation of 6 points, to detect a 5-point difference in the mean GAD-7 score change between the groups (fluoxetine plus digital intervention or fluoxetine alone).

The statistical analyses were performed using the Statistical Software for Social Sciences (SPSS) for Windows, version 29. As our aim was to analyze the sociodemographic characteristic data of participants at baseline, we used the sample descriptive analyses of both clinical studies in this manuscript.

## Results

**Figure 1:**
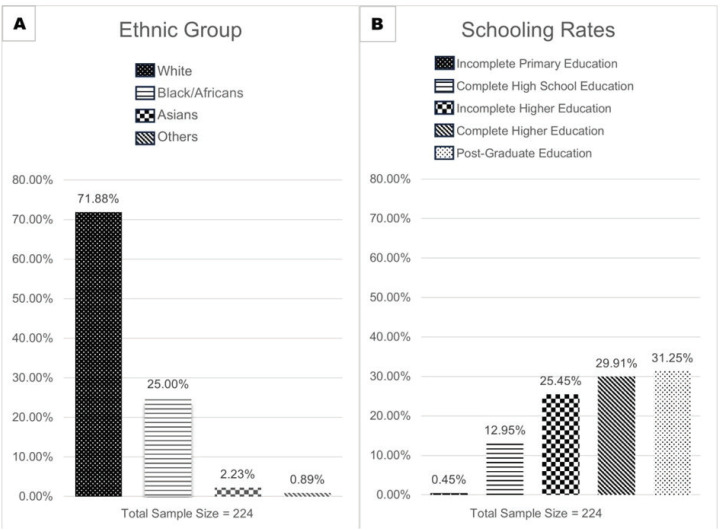
A) Sample distribution by ethnicity; B) sample distribution by level of education.

The total sample consisted of 224 individuals, with a mean age of 32.63 years. Regarding gender distribution, the sample included 213 females (95.1%). Most participants (n = 161, 71.87%) were self-identified as of White ethnicity. In relation to educational attainment, 31.3% held postgraduate degrees, 29.9% had completed undergraduate studies, and 25.4% had incomplete undergraduate education, with an average of 15.12 years of schooling (Figure 1).

Our analyses shed light on a distinctive profile among patients demonstrating both access to and interest in utilizing digital interventions in the field of psychiatry. These individuals typically belong to a younger age group, identify as White ethnically, possess a higher level of education and are predominantly female. Notably, the predominance of females aligns with established trends, indicating a greater inclination among women to seek treatment for psychiatric disorders. Remarkably, our sample exhibits a significantly higher average of 15.12 years of schooling, surpassing Brazil's national average of 10.1 years.^5^ This substantial educational discrepancy underscores concerns about equitable access to digital therapeutic solutions. Furthermore, when contrasting the prevalence of White individuals in our study population (71.87%) with the national average of 42.8%,^6^ it becomes evident that certain demographic groups might have more extensive access to digital interventions. This disparity highlights the need for a closer examination of how we can improve accessibility for digitally vulnerable populations.

## Discussion

Both studies included in our analyses recruited participants through disclosure in digital media, including social networks of related institutions or personal social networks. Therefore, it is assumed that our sample might have been influenced by the limited reach inherent in this mode of dissemination. The public reached through this disclosure likely consists of individuals who are more inclined towards health interests, actively pursuing medical assistance, and having convenient access to technology services. This poses a significant question about the specific demographic section we actively engage within our mental health studies, particularly those conducted in the digital realm. This prompts us to contemplate the potential bias in our sample selection as it may primarily represent individuals who are more tech-savvy, health-conscious, and proactive in seeking medical support in agreement with other digital psychiatry studies published in the scientific literature.^7-10^ Graham et al.,^11^ conducted a study using mobile app platform for the treatment of Depression and Anxiety in 2020. Their sample also consisted predominantly of individuals of White ethnicity, comprising 65% of the participants. Additionally, a significant majority of the sample had pursued education beyond high school, with 92% having completed college or attained a higher level of education.

Scientific literature demonstrates that the prevalence of mental disorders is higher among non-white and low-educated populations in Brazil,^12-14^ further emphasizing the need to include these individuals in studies and practice of digital psychiatry. However, this population often faces difficulties related to access and digital literacy, which can further exacerbate their challenges in accessing care. Consequently, as digital therapeutic solutions continue to advance, it is crucial not only to assess their efficacy in positive treatment outcomes but also scrutinize their ability to inclusively serve vulnerable and underserved individuals. It is essential to acknowledge that access to the internet is considered a fundamental human right by several countries and organizations, including the United Nations. However, socioeconomic barriers, such as the lack of universal access to electricity in some regions, hinder this right from being fully realized. Therefore, the Government plays an important role in establishing and implementing policies that ensure these rights. Initiatives such as early implementation of digital education in schools and offering fiscal incentives to enhance access to technological resources, can contribute to promote digital inclusion among vulnerable populations.

Additionally, it is imperative to reassess our approach to participant recruitment for research studies. Relying solely on digital means for recruitment may inadvertently exclude digitally vulnerable populations. Exploring traditional advertising methods like newspapers or radio can be instrumental in reaching these segments of the population more effectively. By adopting these measures, we can improve accessibility, benefiting vulnerable individuals and broadening the scope of digital psychiatry. This multifaceted approach might ensure that advancements in digital mental health care are not only effective but also inclusive and accessible to all.

## Data Availability

The data that support this study are available from the authors upon request.
